# Confidence interval of percentiles in skewed distribution: The importance of the actual coverage probability in practical quality applications for laboratory medicine

**DOI:** 10.11613/BM.2019.030101

**Published:** 2019-10-15

**Authors:** Cristiano Ialongo

**Affiliations:** Department of Physiology and Pharmacology, Sapienza University of Rome, Rome, Italy

**Keywords:** biostatistics, confidence intervals, statistical data analysis, health care quality indicators

## Abstract

**Introduction:**

Quality indicators (QI) based on percentiles are widely used for managing quality in laboratory medicine nowadays. Due to their statistical nature, their estimation is affected by sampling so they should be always presented together with the confidence interval (CI). Since no methodological recommendation has been issued to date, our aim was investigating the suitability of the parametric method (LP-CI), the non-parametric binomial (NP-CI) and bootstrap (BCa-CI) procedures for the CI estimation of 2.5^th^, 25^th^, 50^th^, 75^th^ and 97.5^th^ percentile in skewed sets of data.

**Materials and methods:**

Skewness was reproduced by numeric simulation of a lognormal distribution in order to have samples with different right-tailing (moderate, heavy and very heavy) and size (20, 60 and 120). Performance was assessed with respect to the actual coverage probability (ACP, accuracy) against the confidence level of 1-α with α = 0.5, and the median interval length (MIL, precision).

**Results:**

The parametric method was accurate for sample size N ≥ 20 whereas both NP-CI and BCa-CI required N ≥ 60. However, for extreme percentiles of heavily right-tailed data, the required sample size increased to 60 and 120 units respectively. A case study also demonstrated the possibility to estimate the ACP from a single sample of real-life laboratory data.

**Conclusions:**

No method should be applied blindly to the estimation of CI, especially in small-sized and skewed samples. To this end, the accuracy of the method should be investigated through a numeric simulation that reproduces the same conditions of the real-life sample.

## Introduction

The statistical estimation consists of quantifying the true characteristic of a population or phenomenon basing on a limited set of observations. Notably, for the operation of collecting data is indeed a random process of sampling, the estimate is not unique since it may vary depending on the scatter of the sample. The unavoidable uncertainty that estimation carries in can be made explicit by translating the sampling error into a probability distribution (*1*). Thereby, the most extreme variation of the point estimate that is likely to occur can be turned into a pair of values bounding an amount of probabilities. This interval allows the acceptance of any size of estimate variation lying within it and is termed confidence interval (CI), to which in turn corresponds to a probabilistic confidence level (*1*).

By the perspective of sampling, α out of 100 equally sized samples withdrawn under same conditions from the same population (or set of data obtained for the same phenomenon) are expected to give by chance the CI that does not include the true (population) value. This probability corresponds to α or Type I error or the false-positive rate, and it is nothing but the probability to make an untrue statement about the population basing on the sample estimate (*1*). Mathematically, the confidence level is defined as 100-α or (1-α)·100%.

In practice, the number of times the CI complies with the confidence level corresponds to the actual coverage probability (ACP), and represents the characteristic performance of the CI (2). For CI bounds are estimates themselves and thus affected by the sampling error, it turns out that the declared confidence level may not coincide with the one actually observed. Therefore, a reliable CI method is the one of which ACP closely approaches the stated confidence level (*3*).

In the exercise of quality it is a common practice using point estimates (*2*). In this regard, laboratory medicine has shown since the 1980s a significant interest for the percentile-based quality indicator (QI), particularly for it can suit well both internal and external assessment of quality and proficiency. In the internal management of quality, percentile-based QIs have been introduced to gauge the timeliness of sample testing (*4*). For instance, the point estimate of the 50^th^ and 90^th^ percentile of the laboratory turnaround time (TAT) has been used to investigate the performance change after an intervention or to compare the actual performance with a pre-established quality goal (*5*). By contrast, in external quality assessment based on participatory exercises or surveys, percentile-based QIs have been adopted to provide factual quality goals basing on the distribution of the participants’ score according to the “state-of-the-art” principle (*6*, *7*). In this case, the 25^th^, 50^th^ and 75^th^ percentile have been naturally adopted since suiting well the representation of quality ladder (*e.g.* “poor”, “adequate” and “optimal” respectively) (*8*).

Despite the use of percentile-based QIs is broadly adopted by official organs of laboratory medicine like the International Federation of Clinical Chemistry (IFCC), actually we do not observe the same methodological attention that has been devoted to the reference interval (RI) that shares the same statistical nature (*9*, *10*). Therefore, to date there is no official recommendation on the use of the CI for percentile-based QIs. In order to support and promote the use of CI for this kind of indicators, we have investigated the reliability of methods for CI estimation in skewed and relatively small sized samples, a condition often encountered in quality data analysis. Particularly, we have investigated the characteristic performance of one parametric method based on lognormal transformation (LP-CI), and of two non-parametric procedures respectively based on the binomial partition of the quantiles (NP-CI) and the bias corrected-accelerated bootstrap (BCa-CI). Moreover, a simple case study has been carried out in order to show how the methodology used in this work can provide the CI reliability in a single sample of real-life data, and how this would impact on the conformity assessment to quality requirements.

## Materials and methods

### The CI estimation

For the principles behind the methods used in this study have been already discussed extensively, in this section it will be given only a very brief presentation (*11*).

**Figure 1:**
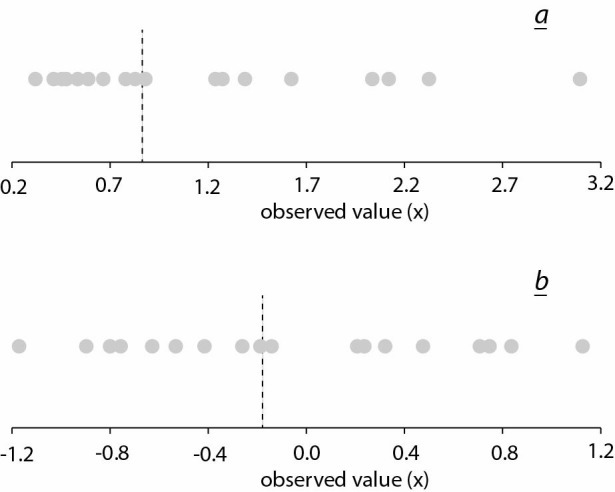
Effect of transformation on order statistics. Data in panel “a” are lognormally distributed and the vertical line marks the median; when the log-transformation is applied as shown in panel “b”, relative distances change and data re-distributes according to a Gaussian-shape; it can be seen that the transformation does not affect the partition ratio since the number of dots on each side of the median remains the same, so that the transformation affects only the scale in which the percentile is represented.

The parametric method – since it was devised for fairly normal datasets, estimation of CI bounds by the LP-CI depends on data transformation. Thus, recalling that the percentile is statistic that depends on the order of a series of points x_i_, y_i_ = g(x_i_) is a suitable transformation if it does not change the order but affects only the relative distances within the dataset so that y_i_ is normally distributed as shown in Figure 1. Thereby, the CI bounds can be estimated on y_i_ and then back-transformed to x_i_ by means of the function x_i_ = g^-1^(y_i_). For instance, if g is the natural logarithm, then g^-1^ is the antilog or base-e exponential (*12*, *13*).

The non-parametric procedures – in this place it will be only recalled that the percentile is a partition point of an ordered data set (*e.g.* 25^th^ percentile = 0.25 or 1:4). Thereby, the binomial distribution can be used to estimate the largest and smallest value within the actual data that the percentile may take because of sampling, as it is done in the NP-CI (11). Alternatively, the same extremes can be found empirically (BCa-CI) by choosing the pair from the frequency distribution of the values that the percentile takes in a large number of re-samples of the actual data (*11*). Notably, whereas the NP-CI relies on a discrete set of values, the BCa-CI is instead from a continuous one, although both of them are constrained within the actual range of observed points.

Equations used for each method in this study are detailed in Table 1 with the relative explanation.

**Table 1:** Equations for bounds of the confidence interval

**Equation of bounds**	**Symbols and notes**
**Lognormal-parametric (LP-CI)**	
**upper = e ^[m – (t1-α/2,[n-1,λ]^^·^^s^^·^^n^-0.5^)]^** **lower = e ^[m – (tα/2,[n-1,λ]^^·^^s^^·^^n^-0.5^)]^**	The e is the base of the natural logarithm (ln); m, s and n are the average, standard deviation and size of the normalized sample, t_1-α/2,[n-1,λ]_ and t_α/2,[n-1,λ]_ are the quantiles of the non-central t distribution with n-1 degrees of freedom and non-centrality parameter λ = -z·n^0.5^ (z is the quantile of the standardized normal distribution corresponding to the percentile of the sample)
**Non-parametric (NP-CI)**	
**upper = (n·q)–z_α/2_·[(n·q)·(1-q)]^0.5^** **lower = (n·q)+z_α/2_·[(n·q)·(1-q)]^0.5^**	The n is the sample size, q is the partition ratio of the quantile (*e.g.* 10^th^ percentile is 0.1) and z_α/2_ is the quantile of the standardized normal distribution function
**Bias corrected-accelerate bootstrap (BCa-CI)**	
**upper = Φ(^z_0_+[(^z_0_+z_α_)·(1-^a·(^z_0_+z_α_)^-1^])** **lower = Φ(^z_0_+[(^z_0_+z_1-α_)·(1^a·(^z_0_+z_1-α_)^-1^])**	The Φ is the cumulative standard normal distribution, z_α_ and z_1-α_ are the quantiles of the standard normal distribution, ^z_0_ and ^a are parameters for the resampling bias and skewness
CI – confidence interval.	

### Simulation study

**Figure 2:**
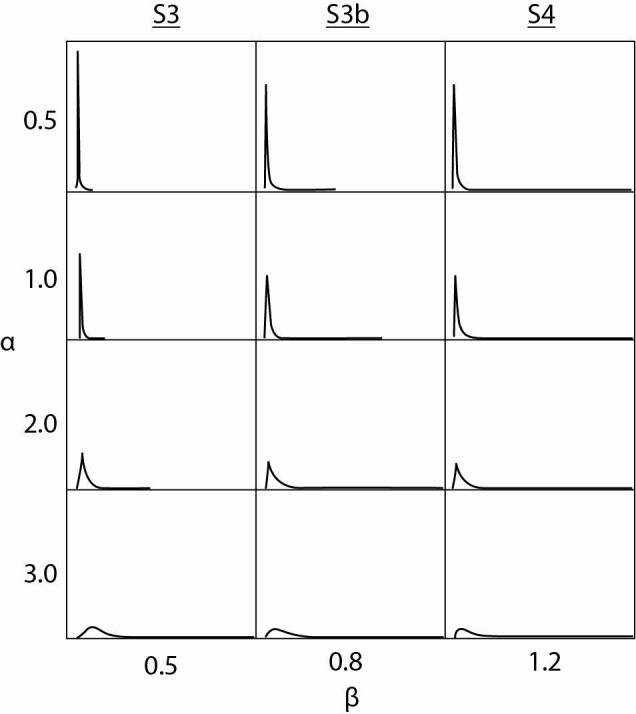
Actual shape of the 3-parameter lognormal probability density function used for generating the artificial samples according to parameters of scale (β) and location (α). The testing conditions described within the result section are S3 (β = 0.5, any α), S3b (β = 0.8, any α) and S4 (β = 1.2, any α); γ (threshold) was set equal to 0 in any simulation allowing only non-null positive values. For each panel, vertical axis was data density and horizontal axis was the random variable X.

A theoretical model represented by the generalized 3-parameter lognormal distribution was used to generate sets of artificial data each featured by a combination of location (α = 0.5, 1.0, 2.0 and 3.0) scale (β = 0.5, 0.8 and 1.2) and threshold (γ = 0) in order to reproduce a particular degree of asymmetry and tailing (*i.e.* skewness) for only positive values (X ≥ 0). Particularly, the combinations of scale and location parameters were chosen so to give rise to the data models as in Figure 2: S3) for β = 0.5 the shape was mildly right-skewed and changed from minimal right-tailed and platykurtic by α= 0.5 to heavily right-tailed and platykurtic by α = 3.0; Figure 2: S3b) for β = 0.8 the shape was heavily skewed with more pronounced right-tailing; Figure 2: S4) for β = 1.2 the shape was very heavily skewed and left-fronted (*i.e.* almost no left tail) turning from leptokurtic with short right-tailing by α = 0.5 to platykurtic with long right-tailing by α = 3.0.

For any possible combination of parameters, it was generated 3 batches of 100 samples sized N = 20, N = 60 and N = 120 respectively, and for each of them the CI was estimated for the 2.5^th^, 25^th^, 50^th^, 75^th^ and 97.5^th^ percentile using the equations shown in Table 1 for LP-CI, NP-CI, and BCa-CI, respectively.

### Accuracy and precision

Accuracy and precision of the CI estimation were represented by respectively the ACP and the median interval length (MIL). Particularly, ACP for each tested condition was obtained by counting the number of estimated CI that contained the true population percentile (calculated whereby the theoretical function generating the samples). The optimum of performance was ACP ≈ 1-α which was set equal to 0.95 or 95% in this study. Median interval length was computed in each subset of 100 artificial samples by taking the median of the differences between the upper and lower bound of the CI. The MIL was reported only when the corresponding ACP was at least > 90%.

All the calculations were performed using Excel 2010 (Microsoft Corp., Redmond, CA), except for BCa that was performed using SPSS 20.0 (IBM Corp., Armonk, NY) and data generation that was carried out exploiting the pseudo-random number generator embedded in Minitab 17 (Minitab Inc., State College, PA).

### Case study

From a very large set of real-life turnaround time (TAT) data used in previously pushed studies on laboratory quality, a subset sized N = 27 of STAT tests requested by the Emergency Department in a single morning shift was selected as it showed right tailing (*5*, *14*). In order to assess whether the laboratory could suite the timeliness required by the Emergency Department, two performance specifications were established and two percentile-based QI namely the MED (50^th^ percentile) and the P90 (90^th^ percentile) were computed accordingly (*15*). Particularly, as quality goal it was stated that MED < 35 minutes and P90 < 55 minutes. The CI reliability under sample conditions was assessed by way of a simulation study, following this general procedure:

1. The lognormal model was fitted to the real-life data
2. Goodness-of-it was assessed using the normal probability plot and the Anderson-Darling statistic
3. The true 50^th^ and 90^th^ percentile were computed using the parameters of the lognormal function
4. Same parameters were used to generate 100 artificial random samples sized N = 27
5. The CI was estimated by way of either LP-CI or NP-CI or BCa-CI
6. The ACP was calculated counting the times the CI contained the true parameter.

The full procedure is detailed in the Supplementary material.

## Results

### CI accuracy

When the shape was the kind of S3 and thus mildly skewed (Table 2) as well as of S3b (Table 3) and thus heavily skewed, the LP-CI resulted to be the best performing method regardless of sample size. In fact, LP-CI was able to provide CI estimates with ACP close to 95% for both central and extreme percentiles. On the contrary, NP-CI as well as BCa-CI were able to give acceptable estimates for extreme percentiles only when N ≥ 60. It must be noted that under some conditions the three methods and particularly NP-CI seemed to be conservative with actual coverage probability about 98-100%, although quite spuriosly. When shape was the kind of S4 and thus very heavily skewed (Table 4), even the LP-CI required N ≥ 60 to reliably estimate the CI bounds for extreme percentiles. A comparable behaviour was observed for both NP-CI and BCa-CI under same conditions by N ≥ 120.

**Table 2:** Performance characteristics of confidence interval estimation with confidence level of 95% under to the lognormal model of skewness (S3)

**β = 0.5**		**N = 20**			**N = 60**			**N = 120**		
		**LP-CI**	**NP-CI**	**BCa-CI**	**LP-CI**	**NP-CI**	**BCa-CI**	**LP-CI**	**NP-CI**	**BCa-CI**
	**Percentile**	**ACCURACY (ACTUAL COVERAGE PROBABILITY, %)**								
**α = 0.5**	2.5^th^	93	31	40	92	81	84	98	87	93
	25^th^	95	93	92	94	93	91	98	95	95
	50^th^	93	91	91	97	98	94	96	98	94
	75^th^	94	98	83	98	98	96	92	92	92
	97.5^th^	94	35	§	96	78	30	92	93	89
**α = 1.0**	2.5^th^	95	30	41	95	75	78	93	88	94
	25^th^	95	93	92	93	95	96	93	93	90
	50^th^	95	96	94	95	96	96	95	97	92
	75^th^	95	94	90	96	96	93	97	98	96
	97.5^th^	96	44	§	99	80	48	97	92	90
**α = 2.0**	2.5^th^	98	36	43	93	70	79	93	88	94
	25^th^	95	96	92	93	94	93	93	92	92
	50^th^	95	91	91	97	96	95	96	96	96
	75^th^	96	98	94	98	98	94	96	94	92
	97.5^th^	97	42	§	94	80	53	96	92	90
**α = 3.0**	2.5^th^	94	32	36	93	76	81	93	91	95
	25^th^	94	94	91	94	96	95	94	93	93
	50^th^	95	93	94	96	98	95	95	93	91
	75^th^	92	95	85	99	98	94	97	93	94
	97.5^th^	94	31	§	99	81	40	95	94	92
**β = 0.5**		**N = 20**			**N = 60**			**N = 120**		
		**LP-CI**	**NP-CI**	**BCa-CI**	**LP-CI**	**NP-CI**	**BCa-CI**	**LP-CI**	**NP-CI**	**BCa-CI**
	**Percentile**	**PRECISION (MEDIAN INTERVAL LENGTH, *arbitrary unit*)**								
**α = 0.5**	2.5^th^	0.46	*	*	0.26	*	*	0.19	*	0.29
	25^th^	0.58	0.84	0.70	0.33	0.39	0.35	0.23	0.29	0.26
	50^th^	0.76	0.79	0.79	0.42	0.56	0.49	0.29	0.38	0.34
	75^th^	1.26	1.54	0.94	0.65	0.85	0.76	0.46	0.52	0.50
	97.5^th^	4.17	*	^§^	2.00	*	*	1.36	2.08	*
**α = 1.0**	2.5^th^	0.76	*	*	0.44	*	*	0.31	*	0.51
	25^th^	0.97	1.30	1.04	0.55	0.75	0.73	0.39	0.33	0.45
	50^th^	1.26	1.32	1.33	0.70	0.91	0.90	0.49	0.62	0.58
	75^th^	2.05	2.77	1.90	1.13	1.50	1.39	0.77	0.90	0.90
	97.5^th^	6.60	*	^§^	3.45	*	*	2.32	3.57	3.26
**α = 2.0**	2.5^th^	2.10	*	*	1.18	*	*	0.85	*	1.32
	25^th^	2.57	3.72	2.84	1.48	1.94	1.65	1.04	1.00	1.24
	50^th^	3.34	3.56	3.56	1.88	2.38	2.26	1.33	1.65	1.56
	75^th^	5.41	6.88	5.41	2.97	4.01	3.34	2.09	2.45	2.36
	97.5^th^	17.82	*	^§^	9.00	8.33	*	6.25	9.34	8.62
**α = 3.0**	2.5^th^	5.62	*	*	3.24	*	*	2.33	3.35	3.83
	25^th^	6.99	9.94	7.99	4.06	5.22	4.71	2.87	3.95	3.30
	50^th^	8.96	9.97	10.08	5.21	6.61	5.82	3.67	4.60	4.44
	75^th^	14.67	18.41	13.31	8.20	10.62	9.16	5.77	6.55	6.55
	97.5^th^	46.48	*	^§^	24.65	*	*	17.31	27.78	26.44
CI - confidence interval. LP-CI - Lognormal-parametric CI. NP-CI - Non-parametric CI. BCa-CI - Bias corrected-accelerated CI. *unreliable value since actual coverage probability below < 90%. ^§^unable to achieve 1000 complete iteration for computing bounds. Lognormal parameters: α=location, β=scale.										

**Table 3:** Performance characteristics of confidence interval estimation with confidence level of 95% under to the lognormal model of skewness (S3b)

**β = 0.8**		**N = 20**			**N = 60**			**N = 120**		
		**LP-CI**	**NP-CI**	**BCa-CI**	**LP-CI**	**NP-CI**	**BCa-CI**	**LP-CI**	**NP-CI**	**BCa-CI**
	**Percentile**	**ACCURACY (ACTUAL COVERAGE PROBABILITY, %)**								
**α = 0.5**	2.5^th^	94	38	49	95	65	73	96	91	92
	25^th^	93	96	91	95	98	98	93	92	95
	50^th^	96	94	98	97	98	93	95	95	94
	75^th^	97	99	91	96	98	97	95	95	95
	97.5^th^	95	32	§	94	80	37	98	95	91
**β = 0.8**		**N = 20**			**N = 60**			**N = 120**		
	**Percentile**	**LP-CI**	**NP-CI**	**BCa-CI**	**LP-CI**	**NP-CI**	**BCa-CI**	**LP-CI**	**NP-CI**	**BCa-CI**
**α = 1.0**	2.5^th^	97	34	38	96	76	79	92	91	96
	25^th^	96	96	80	95	98	97	93	94	92
	50^th^	95	92	93	97	99	99	96	99	97
	75^th^	92	98	69	96	98	87	95	94	92
	97.5^th^	96	34	^§^	93	77	45	92	93	92
**α = 2.0**	25^th^	95	97	93	95	90	90	92	96	97
	50^th^	94	95	90	93	90	91	92	95	93
	75^th^	93	96	94	95	93	90	94	96	97
	97.5^th^	94	49	^§^	95	72	44	95	92	91
**α = 3.0**	2.5^th^	94	19	26	97	66	73	98	94	91
	25^th^	93	94	93	92	95	94	97	96	97
	50^th^	96	95	96	94	96	92	97	95	96
	75^th^	93	96	93	92	93	90	98	94	94
	97.5^th^	94	40	^§^	95	74	48	96	94	91
		**PRECISION (MEDIAN INTERVAL LENGTH, *arbitrary unit*)**								
**α = 0.5**	2.5^th^	0.42	*	*	0.24	*	*	0.17	0.21	0.25
	25^th^	0.79	1.12	1.01	0.44	0.57	0.49	0.30	0.34	0.36
	50^th^	1.27	1.35	1.80	0.68	0.90	0.83	0.48	0.62	0.60
	75^th^	2.53	2.86	2.18	1.35	1.73	1.50	0.92	1.09	1.10
	97.5^th^	12.33	*	^§^	6.09	*	*	4.05	6.01	5.71
**α = 1.0**	2.5^th^	0.67	*	*	0.39	*	*	0.28	0.34	0.38
	25^th^	1.28	1.77	*	0.71	0.91	0.87	0.50	0.68	0.58
	50^th^	2.08	2.24	4.83	1.11	1.34	1.31	0.78	0.94	0.92
	75^th^	4.33	4.75	*	2.12	2.68	2.10	1.50	1.71	1.60
	97.5^th^	22.14	*	^§^	9.68	*	*	6.58	11.26	10.72
**α = 2.0**	2.5^th^	1.87	*	*	1.07	*	*	0.76	0.92	1.13
	25^th^	3.54	4.80	4.34	1.96	2.43	2.15	1.38	1.22	1.77
	50^th^	5.87	5.92	6.03	3.06	4.13	3.50	2.12	2.75	2.45
	75^th^	11.81	15.02	12.52	5.87	7.80	6.31	4.12	4.74	4.67
	97.5^th^	62.97	*	^§^	26.20	*	*	18.20	26.69	26.46
**α = 3.0**	2.5^th^	5.03	*	*	2.87	*	*	2.06	2.39	3.07
	25^th^	8.84	11.31	10.10	5.27	6.77	5.70	3.72	3.52	4.40
	50^th^	13.76	15.85	19.43	8.08	11.16	9.70	5.80	7.65	7.79
	75^th^	27.10	38.34	34.51	15.64	20.18	16.53	11.14	12.31	12.91
	97.5^th^	134.76	*	^§^	71.22	*	*	49.50	74.82	72.09
CI - confidence interval. LP-CI - Lognormal-parametric CI. NP-CI - Non-parametric CI. BCa-CI - Bias corrected-accelerated CI. *unreliable value since actual coverage probability below < 90%. ^§^unable to achieve 1000 complete iteration for computing bounds. Lognormal parameters: α=location, β=scale.										

**Table 4:** Performance characteristics of confidence interval estimation with confidence level of 95% under to the lognormal model of skewness (S4)

**β = 1.2**		**N = 20**			**N = 60**			**N = 120**		
		**LP-CI**	**NP-CI**	**BCa-CI**	**LP-CI**	**NP-CI**	**BCa-CI**	**LP-CI**	**NP-CI**	**BCa-CI**
	**Percentile**	**ACCURACY (ACTUAL COVERAGE PROBABILITY, %)**								
**α = 0.5**	2.5^th^	93	29	42	98	72	78	97	90	93
	25^th^	96	94	91	97	93	94	97	95	95
	50^th^	94	93	94	98	94	96	97	92	93
	75^th^	96	95	90	95	99	97	96	92	94
	97.5^th^	95	43	^§^	97	74	47	96	94	90
**α = 1.0**	2.5^th^	71	40	64	96	82	88	95	84	88
	25^th^	91	86	87	95	96	96	97	96	95
	50^th^	93	91	93	95	96	94	94	96	95
	75^th^	92	97	90	94	98	96	94	92	93
	97.5^th^	84	66	^§^	98	77	44	95	93	91
**α = 2.0**	2.5^th^	74	33	62	95	76	80	92	82	92
	25^th^	93	90	90	96	95	96	95	90	93
	50^th^	96	93	95	95	95	92	94	96	96
	75^th^	90	92	90	96	97	95	96	98	96
	97.5^th^	75	54	^§^	96	82	45	96	96	93
**α = 3.0**	25^th^	90	90	96	93	95	94	95	95	96
	50^th^	96	94	93	95	95	93	94	96	95
	75^th^	94	96	90	98	96	95	95	92	93
	97.5^th^	83	58	^§^	97	76	44	93	93	88
		**PRECISION (MEDIAN INTERVAL LENGTH, *arbitrary unit*)**								
**α = 0.5**	2.5^th^	0.27	*	*	0.16	*	*	0.12	0.14	0.16
	25^th^	0.88	1.08	1.02	0.51	0.65	0.60	0.35	0.31	0.42
	50^th^	1.84	2.07	2.20	1.04	1.33	1.42	0.72	0.89	0.87
	75^th^	5.40	6.86	5.62	2.62	3.57	2.99	1.80	2.10	2.04
	97.5^th^	51.67	*	^§^	20.33	*	*	12.99	22.16	22.02
**α = 1.0**	2.5^th^	*	*	*	0.26	*	*	0.19	*	*
	25^th^	1.53	*	*	0.84	1.08	0.99	0.58	0.48	0.70
	50^th^	4.03	4.07	4.83	1.78	2.11	1.96	1.20	1.57	1.53
	75^th^	13.67	18.01	15.12	4.55	5.61	4.86	3.00	3.44	3.60
	97.5^th^	*	*	^§^	36.63	*	*	22.16	30.76	27.05
**α = 2.0**	2.5^th^	*	*	*	0.75	*	*	0.51	*	0.74
	25^th^	4.15	4.89	5.20	2.21	2.90	2.64	1.58	0.99	1.83
	50^th^	11.20	10.82	13.09	4.73	5.88	5.52	3.24	4.15	3.91
	75^th^	40.06	47.92	39.67	12.17	15.75	13.64	8.15	9.76	3.91
	97.5^th^	*	*	^§^	97.22	*	*	61.56	94.76	85.71
**α = 3.0**	2.5^th^	*	*	*	1.94	*	*	1.43	*	2.03
	25^th^	10.57	12.20	13.76	5.90	7.71	7.43	4.13	5.49	5.26
	50^th^	28.26	29.63	34.36	12.39	15.88	14.20	8.83	10.93	10.68
	75^th^	99.30	121.85	92.59	31.36	39.59	*	22.25	25.35	24.76
	97.5^th^	*	*	^§^	244.23	*	*	170.15	252.03	*
CI - confidence interval. LP-CI - Lognormal-parametric CI. NP-CI - Non-parametric CI. BCa-CI - Bias corrected-accelerated CI. *unreliable value since actual coverage probability below < 90%. ^§^unable to achieve 1000 complete iteration for computing bounds. Lognormal parameters: α=location, β=scale.										

### CI precision

Under any investigated condition LP-CI delivered the smaller MIL. To this regard it must be remarked that also the difference between the MIL of NP-CI and BCa-CI was often negligible.

### Case study

median and P90 were 34.78 and 43.30 minutes respectively thus within the specifications of MED < 35 and P90 < 55. The results relative to CI and their performance characteristic are shown in Table 5. As it can be seen, from a single analysis the LP-CI gave the shortest interval for both the 50th and 90th percentile. However, the NP-CI was the only one to meet the stated confidence level. Accordingly, the NP-CI showed that only the P90 was met indeed since the upper bound of the 50^th^ percentile (37.65 minutes) was greater than the quality goal of 35 minutes.

**Table 5:** Case study results of turnaround time indicators

	**50^th^ percentile (MED)**	**90^th^ percentile (P90)**
**point estimate (minutes)**	34.78^†^	44.30^†^
**95% LP-CI (minutes)^†^**	33.59 to 37.97	40.72 to 48.18
**ACP (%)^§^**	90	80
**MIL (minutes)**	4.2	7.1
**95% NP-CI (minutes)^†^**	32.38 to 37.65	39.09 to 52.19
**ACP (%)^§^**	96	94
**MIL (minutes)**	5.2	12.9
**95% BCa-CI (minutes)^†^**	32.68 to 37.32	41.19 to 49.85
**ACP (%)^§^**	89	83
MIL (minutes)*	3.8	10.8
MED - 50^th^ percentile-based TAT indicator. P90 - 90^th^ percentile-based TAT indicator. ACP - actual coverage probability. MIL - median interval length. CI - confidence interval. LP-CI - Lognormal-parametric CI. NP-CI - Non-parametric CI. BCa-CI - Bias corrected-accelerated CI. ^†^estimated on real-life data with N = 27. ^§^estimated on 100 samples with N = 27.		

## Discussion

In this study we dealt with the analysis of the CI performances applied to the point estimate of the percentiles used as a quality tool. In this regard, our simulation study showed that the ACP was influenced by the size and asymmetry of the sample, as well as by the position of the percentile for which the CI was estimated. As it can be seen by inspecting the Tables from 2 to 4, LP-CI provided the required accuracy already from N ≥ 20 in many of the conditions investigated. Nevertheless its performance degraded significantly for extreme percentiles of samples where right-tailing was more pronounced. This was also observed for the non-parametric procedures although for them the recovery of accuracy required a much larger sample size and sometimes even greater than 120. Hence, non-parametric procedures are preferable when the sample size is adequately large and it is not possible to identify a normalizing transformation that may be effective. On the other hand, if the transformation was known, the parametric method is preferable because it is less affected by the size of the sample and by the partition ratio of the percentile, particularly when this does not fall into the tail of a heavily right-tailed distribution.

This can be explained by recalling that the probability distribution by means of which the CI method finds out the bounds must be able to describe the effect that sampling has on the point estimate. Such a model depends on the way the random factors contributing to the sampling variability are combined each other, and for the LP-CI the NP-CI and the BCa-CI this is indeed a kind of a fairly balanced equilibrium. In fact, all these methods rely on such distributions like the non-central t, the binomial and the bootstrap that are related with the Gaussian and from which they differ just for a slight degree of skewness. However, for extreme percentiles the corresponding high partitioning ratio (*e.g.* the 2.5^th^ percentile is 0.025 or 1:40) gives rise to an unbalanced factor that tends to distort the sampling distribution, since some of the values for the point estimate that fall on the outer side of the true percentile can be only rarely observed. Obviously, such a factor is further magnified by the small sample size as well as by the skewness of the data, since both of them can cause some partition events to be even rarer or at most impossible at observation. Thereby, unless the ordinary probability model is not adjusted for handling rare events (*e.g.* using parametric instead of non-parametric bootstrap), no CI method should be considered “a priori” capable of providing the declared confidence level regardless of sample size, shape and position of the percentile (*3*, *16*).

Indeed, since the ACP depends on factors that can change from sample to sample, the CI estimated in a single dataset does not provide any information on this fundamental performance. Thereby, concerns could arise about the potential limitations to the application of the CI as a quality tool. In fact, one could argue that using the CI may be even more dangerous than not doing it if there was no means to assess its reliability. In this regard, we used a case study to show that information on the accuracy of the CI under conditions comparable to those of the real-life sample could be obtained through a simple and reproducible simulation procedure.

In particular, the case study concerned the use of the percentile as QI and the comparison of its point estimate in the sample of laboratory data with an arbitrary quality goal. This is a fairly common case, where QI is used to compare the efficiency of a certain laboratory service with the needs or expectations of hospital departments (*17*). Notably, the procedure not only allowed us to demonstrate which method was reliable (namely the NP-CI), but also that the use of the interval instead of the point estimate had a significant impact on the decision-making process. In fact, since the CI was not entirely within the cut-off marking to the quality goal, it was possible to conclude that the judgment of compliance to the specification for the MED (as previously obtained through the simple point estimate) was instead an effect of sampling. Despite this may seem puzzling, owing to the use of the CI we were able to assert that an erroneous judgment (in our case an untrue state of compliance) could only be obtained in 1 out of 20 repetitions of the same quality exercise under the same conditions.

**Figure 3:**
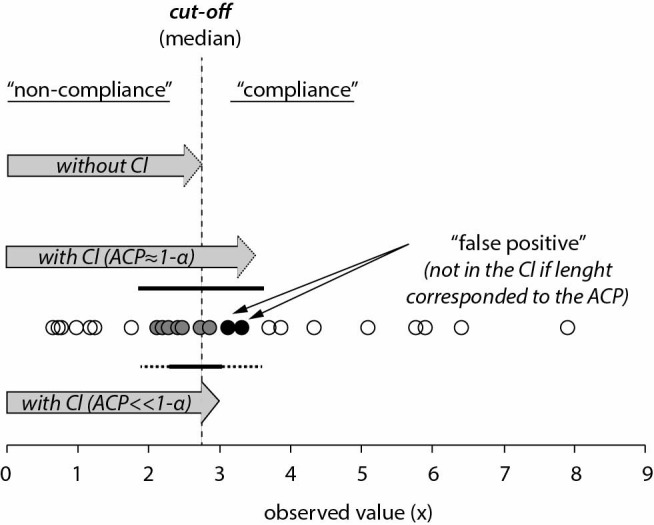
Effect of the actual coverage probability (ACP) of the confidence interval (CI) used to enhance the percentile-based cut-off in a participatory quality exercise. The vertical solid line represents the cut-off established on the median (50^th^ percentile) score of the participants and respect to which it is stated the compliance or not to the performance specification; the application of the CI (solid horizontal line) shifts forward the cut-off to the point of maximum possible variation under the effect of sampling; when the ACP fails to meet the declared level of confidence (*i.e.* ACP << 1-α) there are some of the scores (dark dots) falling inappropriately within the cut-off (dotted horizontal line) that represent kind of false-positives to this exercise.

For the sake of completeness, it should be noted that the procedure outlined in the case study is also suitable when the percentile is used to define the quality goal in a participatory exercise. In fact, the sample variability of the percentile of the distribution of scores is made up by pooling the sample variability of each participant, so it can be used to construct the CI. In this way, the CI shifts the cut-off and modifies some of the judgments on the compliance status, as shown in Figure 3. Assuming that 1-α has been reached, α can be used to indicate the probability of false positivity to the exercise, which gives a measure of the strength of the recommendations to improve or consolidate quality. Remarkably, if the CI were inaccurate, α would be inflated because some of the scores that fall within the interval would instead be found outside the length corresponding to the actual ACP.

Limitations of this study concern the nature of the numeric simulation and are reassumed in the following. Firstly, just some particular combinations of sample size, skewness and position of the percentile were assessed. Hence, there may be some other conditions which can affect differently the ACP of a particular method, as for instance it was shown by Table 5 reporting the results of the case study in real-life samples. Secondly, the ACP provided here is indeed an estimate taken on 100 samples, and thus it is an approximation to the value that would be obtained for convergence taking 1000 or more samples (*3*). Therefore, if this study was replicated generating new data, slight but non-significant differences could be observed. Thirdly, the lognormal model was used just for convenience since the logarithmic transformation is well known and readily understandable. Nonetheless, thus other right-tailed distributions could fit equally well the data in real-life samples. However, because of the scattering caused by sampling (or by the random data generation as in our case), this makes no significant difference in estimation of percentiles and consequently of the CI bounds unless sample size is large enough (*i.e.* N > 500) (*18*). Therefore, although generalizable, results of this study should be used to orient the choice of the CI method basing on the features of the data, and not as definitve proof of its performance.

In conclusion, as no point estimate of percentile should be provided without the CI, especially when it is used as a quality tool in the decision-making process, it is advisable to assess every time the effect of such factors like sample size, skewness and position of the percentile on the method accuracy before applying it. This may be done either by retrieving evidences from literature, either by assessing it directly through a numeric simulation that reproduces the same conditions of the real-life sample. To this end, a procedure like the one used in this study should be adopted to find out the ACP delivered by the method. Of course, the use of numerical simulation would strength the application of percentile-based QI in laboratory medicine.

## Supplementary material

Supplementary material
