# Supplementary material for: Confidence interval of percentiles in skewed distribution: The importance of the actual coverage probability in practical quality applications for laboratory medicine
Source: Biochem Med (Zagreb). 2019 Oct 15;29(3):030101. doi: 10.11613/BM.2019.030101 (PMC6784425; doi:10.11613/BM.2019.030101)
Supplement: Supplementary file 1 — Supplementary material [file bm-29-3-030101-S1.pdf]

Supplementary material

CASE STUDY: Confidence interval (CI) of percentile based quality indicator (QI) for the assessment of timeliness

Part I – CI estimation

The aim of this procedure is to assess whether the TAT of the morning shift for given STAT tests was compliant with internal performance specifications that were set in agreement with clinicians of the Emergency Department, and which were at least 50% of the results delivered within 35 minutes (MED < 35) and at least 90% within 55 minutes (P90 < 55).

The dataset to compute the TAT indicators is the following (in minutes):

35.49 ; 32.23 ; 30.33 ; 28.26 ; 52.19 ; 30.25 ; 43.84 ; 40.31 ; 37.86 ; 33.14 ; 30.84 ; 37.01 ; 33.45 ; 32.68 ; 39.09 ; 32.96 ; 29.87 ; 37.65 ; 32.33 ; 40.53 ; 32.28 ; 34.78 ; 31.54 ; 37.32 ; 44.92 ; 35.59 ; 49.27

By calculation using a simple Excel spreadsheet the 50th and 90th percentiles are 34.78 minutes and 44.3 minutes respectively. Thereby one may conclude that quality goals were met. However, what is the strength of this evidence against the sampling variability? This is not explicit since the percentiles are point estimates and thus subject to imprecision, so the CI is suitable to show this out.

First step is assessing the nature of the distribution of the data. It can be easily achieved plotting both histogram and the percentiles (in this case using the “HISTOGRAM” and the “PROBABILITY PLOT” procedures of Minitab 17):

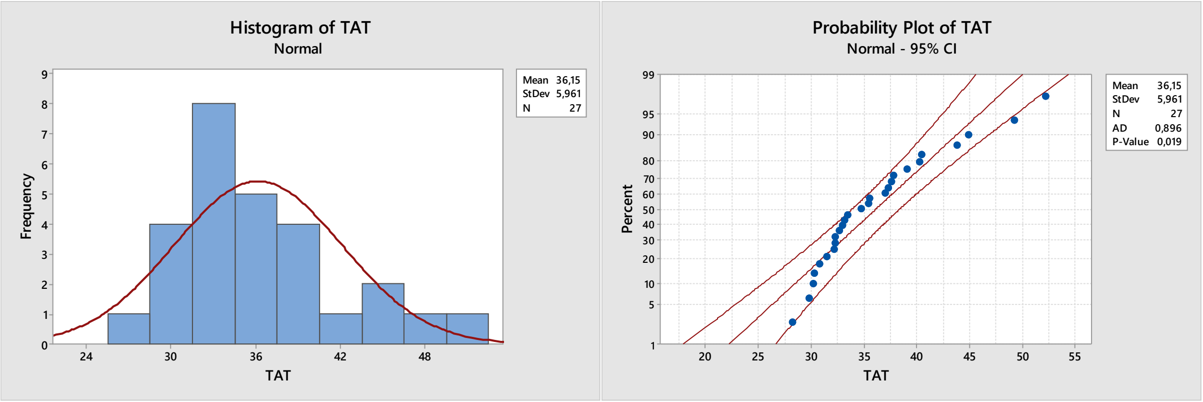

Both the plots show that the data have poor fitting to normal distribution. The Anderson-Darling statistics, which is a measure of fit, is 0.896 and the test shows significant departure from normality with $p < 0.05$. Thereby, in order to apply the parametric method, data must be normalized but for the LP-CI the transformation must be logarithmic. The plots below show the normal fit of the data after transformation:

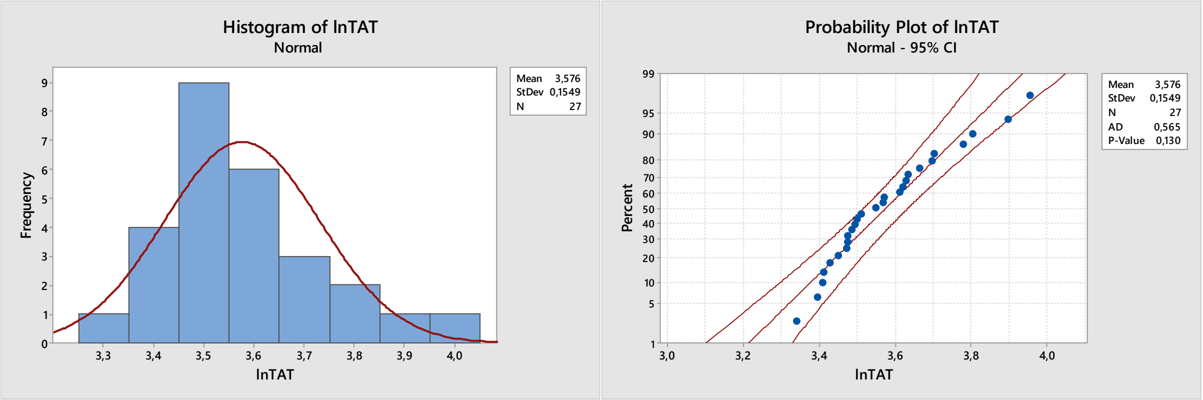

The data look still a bit skewed, however the normality plot shows smaller AD statistic (0.565, better fit) and non-significant departure from normality at the test ($p > 0.05$). Thereby one may attempt to apply the LP-CI procedure and thus to take the lognormal as the reference model for this data.

To compute the LP-CI, one has to find firstly the non-centrality parameter (λ) of the non-central t-distribution, which depends on the sample size (n) and the percentile. The formula to find λ is:

$$\Lambda = -z*n^{0.5}$$

Thus, since z is the quantile of the standardized normal distribution which corresponds to the percentile of the data, z = 0 for the 50th percentile and z = 1.2816 for the 90th percentile. Thus, $\lambda = - 0*(27)^{0.5} = 0$ for the 50th percentile and $\lambda = -1.2816*(27)^{0.5} = -6.6591$ for the 90th percentile. At this point, it is possible to find out the 2.5th and 97.5th percentiles of the non-central t-distribution with given λ and n – 1 = 26 degrees of freedom, since these points represent the extremes of the sampling error in this model for 95% confidence level. The values, computed by means of the on-line calculator Casio’s Keisan, are in the following table:

percentile	z	λ	2.5th $t_{\alpha/2,[n-1,\lambda]}$	97.5th $t_{1-\alpha/2,[n-1,\lambda]}$
0.5	0.00000	0.0000	-2.0555	2.0555
0.9	1.28155	-6.6591	-10.0415	-4.3976

Below it is the web page of Keisan (https://keisan.casio.com/exec/system/1180573220)

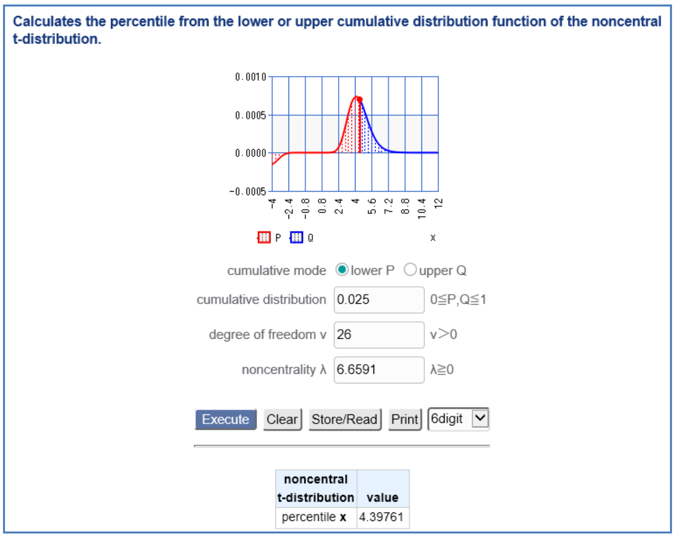

With the average m and standard deviation s of the log-transformed data, the CI boundaries of the 50th percentile can be then computed:

Lower LP-CI bound = $e^{[m - (t_{1-\alpha/2,[n-1,\lambda]}*s*n^{-0.5})]} = e^{[3.5756-(2.0555*0.1549*0.1925)} = 3.5143$

Upper LP-CI bound = $e^{[m - (t_{\alpha/2,[n-1,\lambda]}*s*n^{-0.5})]} = e^{[3.5756-(-2.0555*0.1549*0.1925)} = 3.6369$

And afterwards the CI for the 90th percentile:

Lower LP-CI bound = $e^{[m - (t_{1-\alpha/2,[n-1,\lambda]}*s*n^{-0.5})]}$ = $e^{[3.5756-(-4.3976*0.1549*0.1925)}$ = 3.7067

Upper LP-CI bound = $e^{[m - (t_{\alpha/2,[n-1,\lambda]}*s*n^{-0.5})]}$ = $e^{[3.5756-(-10.0415*0.1549*0.1925)}$ = 3.8750

After back-transformation with exponential function the CI for the 50th and 90th percentile are 33.59 to 37.97 minutes and 40.72 to 48.18 minutes respectively. Except for the percentiles of the non-central t distribution, all calculations can be performed with a simple electronic spreadsheet.

For the non-parametric NP-CI method the calculations are far easier for what is required is the proportion of the percentile p, the sample size n and the quantile of the standardized normal distribution that corresponds to the 97.5th (which is 1.96). Thereby, for the 50th percentile it is:

Lower NP-CI bound = $0.5*27-(1.96*0.5*(1-0.5))^{-0.5}$ = 8

Upper NP-CI bound = $0.5*27+(1.96*0.5*(1-0.5))^{-0.5}$ = 19

And for the 90th percentile it is:

Lower NP-CI bound = $0.9*27-(1.96*0.9*(1-0.9))^{-0.5}$ = 21

Upper NP-CI bound = $0.9*27+(1.96*0.9*(1-0.9))^{-0.5}$ = 27

Recalling that the results of these equations correspond to the partition point of the sample, it is necessary to order the data and picking them up accordingly as follows:

order	***value***	***order***	***value***	***order***	***value***	***order***	***value***	***order***	***value***	***order***	***value***
1	28.26	*6*	31.54	*11*	32.96	*16*	35.59	*21*	39.09	*26*	49.27
2	29.87	*7*	32.23	*12*	33.14	*17*	37.01	*22*	40.31	*27*	52.19
3	30.25	*8*	32.28	*13*	33.45	*18*	37.32	*23*	40.53		
4	30.33	*9*	32.33	*14*	34.78	*19*	37.65	*24*	43.84		
5	30.84	*10*	32.68	*15*	35.49	*20*	37.86	*25*	44.92		

Thereby the CI for the 50th and 90th percentile is 32.28 to 37.65 minutes and 39.09 to 52.19 minutes respectively. Also, in this case, a simple electronic spreadsheet is sufficient.

In order to apply the BCa-CI it is necessary to perform resampling and estimate the parameters of the bootstrap distribution, thereby it is suitable to use a software package that automates operation (like SPSS). According to the results of computation (not shown) the CI for the 50th and 90th percentile are 32.68 to 37.32 minutes and 41.19 to 49.85 minutes respectively.

Part II – Actual coverage probability (ACP) estimation

In order to provide a reliable estimate of the CI, the ACP of the method must be as close as possible to the stated confidence level. A simulation study is required to assess this, and to this end it is necessary to assume that data follow a distribution model in order to find out the true value of the percentiles that are estimated by means of the sample data.

As it was shown previously, the dataset shows good agreement with the lognormal model for log-transformed data were fairly normally distributed:

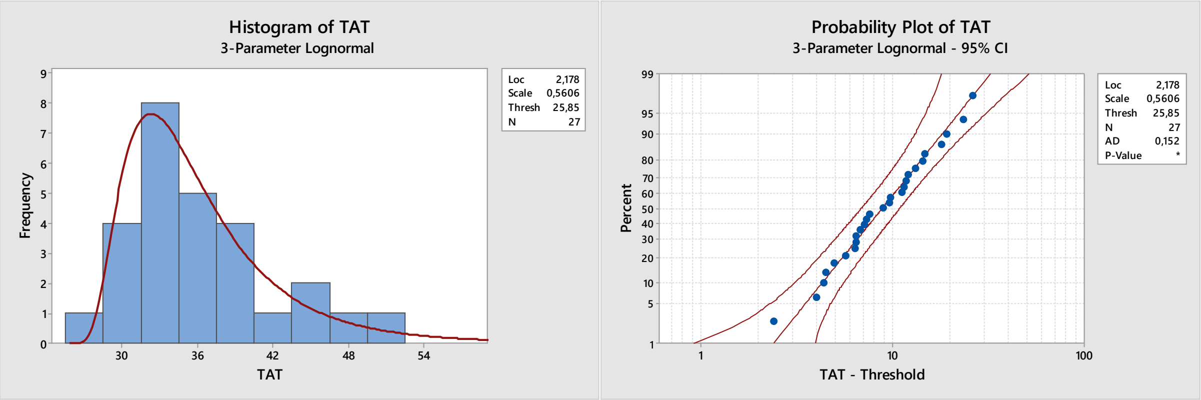

The goodness of the fit is shown by the AD statistic and is 0.152. For comparison, the AD for other right-tailed distributions, namely the log-logistic and the Weibull is 0.172 and 0.175, respectively. To this regard, one should bear in mind this does not mean that necessarily the data come from a lognormal distribution, rather that this model well approximates the putative originating population.

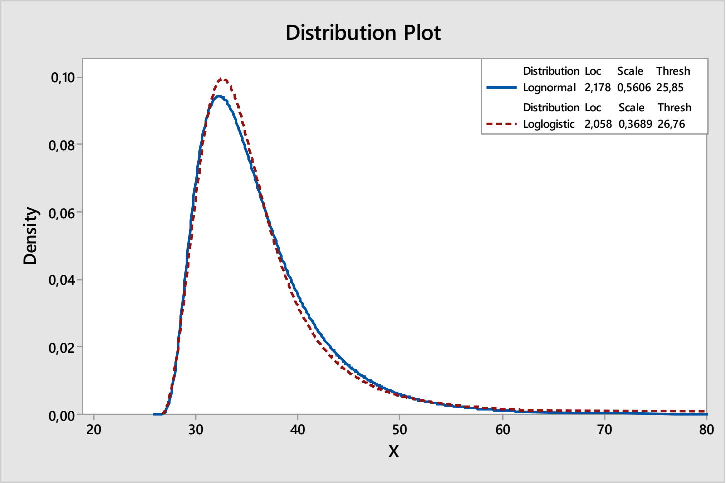

The plot above shows the trivial difference between the lognormal and the log-logistic models fitting the actual dataset. The lognormal parameters used to perform the simulation study are thus α = 2.178, β = 0.5606 and γ = 25.85.

To find out the percentiles of the lognormal distribution with parameters α, β and γ it is possible to use software packages, however an “empirical” method allows achieve suitable approximation avoiding cumbersome calculations. It relies on a simulation where at least three huge samples (N > 1,000,000) are generated and percentiles of interest are calculated on each of them. Thereby, the approximation of the “true” percentile is the average of the point estimates of the sample percentile of three samples sized N = 50,000,000 each. The calculated percentile obtained by means of the cumulative distribution function is thus:

	**Percentile**						
**sample**	2.5th	10th	25th	50th	75th	90th	97.5th
**1**	28.7928	30.1534	31.8978	34.6781	38.7363	43.9612	52.3391
**2**	28.7924	30.1539	31.8987	34.6778	38.7351	43.9569	52.3289
**3**	28.7924	30.1548	31.8997	34.6787	38.7359	43.9631	52.3494
**Calculated**	28.792	30.154	31.899	34.679	38.736	43.960	52.340
**Mean**	28.793	30.154	31.899	34.678	38.736	43.960	52.340
**CV%**	0.0013	0.0024	0.0029	0.0014	0.0016	0.0073	0.0196
**Bias**	-0.001	0.000	0.000	0.001	0.000	0.000	0.000

As it can be seen, the difference falls by the third decimal place and this has no reasonable impact on the assessment of the ACP. Thus, calculations can be carried out using an electronic spreadsheet for arranging an array of artificial samples.

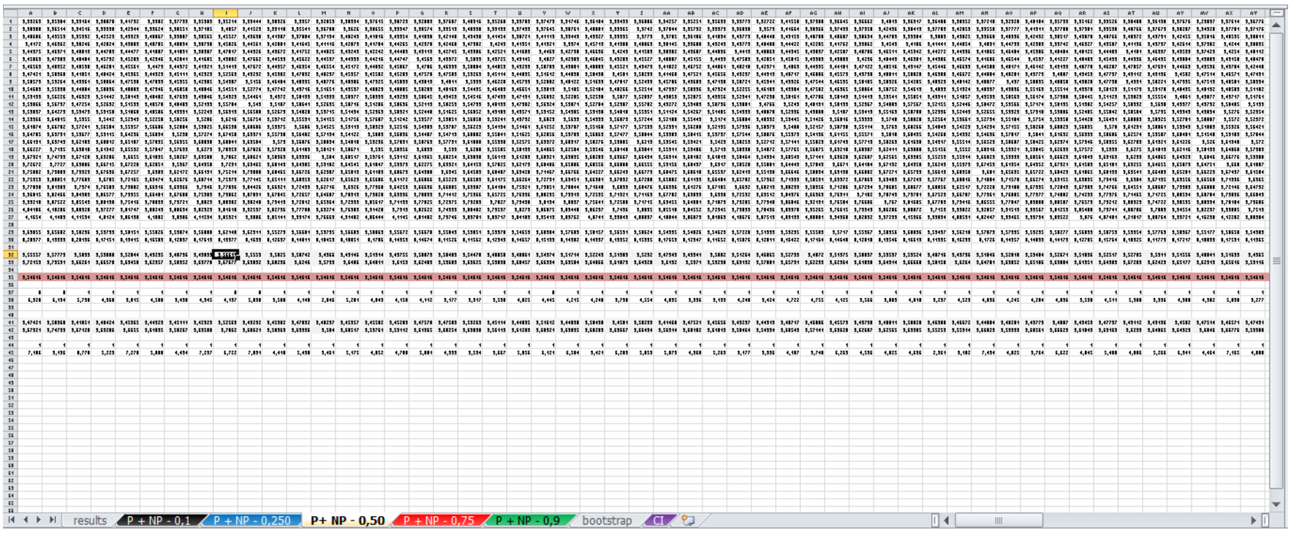

The results are summarized in the following table:

	ACP (%)		
**p.tile**	LP-CI	NP-CI	BCa-CI
**10th**	95	91	95
**75th**	98	96	90
**50th**	90	96	89
**75th**	90	95	83
**90th**	80	94	84

This shows that no method except for the NP-CI was capable of delivering reliable CI estimate under the sample's conditions. To this concern some considerations are mandatory. Firstly, the performance of the LP-CI method depends on the goodness of the normalizing transformation, and indeed recalling what seen earlier, the lognormal was not optimum for it did not seem reduce completely the right tailing. In fact, if the appropriate transformation was used (*i.e.* the exponential ^-2.5), the ACP would be instead:

ACP (%) of LP-CI with transformation ^-2.5				
**10th**	25th	50th	75th	90th
**97**	96	96	95	97

This fact raises the second point of discussion that concerns the reliability of the ACP with respect to the fitted model. There is panoply of non-Gaussian distributions which can produce heavy right-tailing, and many of them can fit the actual data as it is shown below:

		**Percentile**						
**Distribution (paramters)**	**AD**	**2.5th**	**10th**	**25th**	**50th**	**75th**	**90th**	**97.5th**
Lognormal (3)	0.152	28.793	30.154	31.899	34.678	38.736	43.960	52.339
Weibull (3)	0.175	28.689	29.831	31.702	34.890	39.264	44.149	50.574
Log-logistic (3)	0.172	28.787	30.241	31.980	34.590	38.503	44.373	57.012
Fatigue Life (3)	0.149	28.803	30.113	31.856	34.709	38.874	44.043	51.698
Inverse Gaussian (3)	0.149	28.804	30.132	31.872	34.695	38.826	44.013	51.843
Frechet (3)	0.167	28.722	30.220	31.978	34.649	38.542	44.792	53.038
Dagum (4)	0.167	28.732	30.222	31.976	34.638	38.528	43.799	53.151
Burr (4)	0.176	28.711	29.863	31.732	34,900	39.253	44.159	50.718
Gamma (3)	0.168	28.756	29.985	31.797	34.812	39.065	44.076	51.107

However, for they are closely related and data are scattered by sampling, it is impossible to find an “exact” fit. The AD statistics shows that there are at least 9 distributions fitting the data adequately, and none of them produces a significant change in the calculation of the true percentiles. Therefore, the aim of this procedure is not finding the “true” match between the data and a theoretical model, but to find a suitable model to handle them. At this stage, we are not interested in whether the data are lognormal or log-logistic, but if the CI method can be applied with reliability. For instance, we herein attempted the lognormal one for it allows use the LP-CI method, nonetheless the TAT is known to follow the log-logistic distribution as it has been shown in large set of data. Hence, at the sample level

there is no means to discriminate perfectly between shapes and one can be confident of any of those models giving comparably low AD statistics.
